# The Near Point of Convergence in Patients with Vestibular Migraine

**DOI:** 10.1055/s-0044-1791643

**Published:** 2025-02-05

**Authors:** Francisco Carlos Zuma e Maia, Bernardo Faria Ramos, Roseli Saraiva Moreira Bittar, Renato Valerio Rodrigues Cal, Leonel Almeida Luís, Pedro Luiz Mangabeira Albernaz

**Affiliations:** 1 Department of Otorhinolaryngology, Clínica Maia, Canoas, RS, Brazil; 2 Department of Otorhinolaryngology, Universidade Federal do Espírito Santo, Vitória, ES, Brazil; 3 Department of Otoneurology, Hospital das Clínicas da Universidade de São Paulo (HCFMUSP), São Paulo, SP, Brazil; 4 Department of Otorhinolaryngology, Centro Universitário do Estado do Pará (CESUPA), Belém, PA, Brazil; 5 Department of Otorhinolaryngology, Centro Hospitalar Universitário Lisboa Norte EPE, Lisboa, Portugal; 6 Department of Otorhinolaryngology, Faculty of Medicine, Universidade de Lisboa, Lisboa, Portugal; 7 Department of Otorhinolaryngology, Hospital Israelita Albert Einstein, São Paulo, SP, Brazil

**Keywords:** migraine disorders, near point of convergence, convergence insufficiency, biomarkers

## Abstract

**Introduction** Vestibular migraine (VM) is one of the most common vestibular disorders and its diagnosis is based entirely on clinical features. A recent case series suggested a possible link between migraines and convergence insufficiency.

**Objective** To compare the near point of convergence (NPC) in patients with and without VM.

**Methods** We retrospectively reviewed the data of 50 patients with and 50 without VM, comparing the NPC between both groups. The NPC was measured according to the recommendations of the American Academy of Ophthalmology. Differences in the results between groups were compared using the Mann-Whitney test. The association of the NPC with age, gender, and the use of corrective glasses or contact lenses was evaluated by the Student *t* or Mann-Whitney tests for parametric and nonparametric data, respectively. To determine the diagnostic accuracy and optimal cut-off point, receiver operating characteristic (ROC) curves were created.

**Results** The mean NPC was significantly higher in patients with VM (18.50 ± 5.88 cm) compared to the control group (8.06 ± 1.46 cm; *p* < 0.001). The area under the curve (AUC) was 0.986 (95% CI: 0.938–0.999; *p* < 0.0001), suggesting that NPC was able to accurately discriminate between patients with and without VM with a sensitivity of 94% and specificity of 100%.

**Conclusion** Our results suggest that convergence insufficiency is a common sign in patients with VM and may be considered a potential clinical biomarker. However, further studies are needed to confirm this hypothesis.

## Introduction

Vestibular migraine (VM) is one of the most common vestibular disorders worldwide, known to affect 1 to 2.7% of the general population. 1 2 3 It is diagnosed based on a history of migraine, recurrent vestibular symptoms, a temporal association between migraine and vestibular symptoms, and exclusion of other causes of vestibular symptoms. 2 Therefore, the diagnosis is based entirely on clinical features reported by the patient.

A case series suggested a possible link between migraine and convergence insufficiency. 4 The authors also recommended that clinicians seek a history of migraine in patients complaining of reading difficulties secondary to new-onset convergence insufficiency, proposing a prospective study to confirm the possible association between these clinical features.

The near point of convergence (NPC) is an easy-to-perform, standard test that does not require special equipment. It is defined as a measurement of the point where the visual axes intersect under the maximum effort of the convergence. The aim of this study was to compare the NPC in patients with and without (controls) VM.

## Methods

We retrospectively reviewed the data of 50 patients with and 50 without VM, comparing the NPC between both groups. The participants were consecutively selected in order of appearance and allocated between the two groups based on the presence or absence of the clinical criteria for vestibular migraine.

Ethical approval was waived by the local Ethics Committee of the Lutheran University of Brazil in view of the study's retrospective nature and all the procedures being performed as part of routine care.

The NPC was measured according to the American Academy of Ophthalmology's recommendations. 5 A fixation target (examiner's finger) and a ruler were used as required tools to perform the test. In the seated position, patients should look directly at the fixation object that is held in the midsagittal plane, approximately 50 cm away from their head. The examiner moves the fixation object slowly and smoothly in the midsagittal plane closer to patients' nose. When the NPC is reached, the examiner moves the fixation object away from patients' eyes in the midsagittal plane until they start to fixate on the object with both eyes. The room should be well illuminated so that the examiner can notice any changes in eye movements.

Differences in the NPC between the two groups were compared using the Mann-Whitney test. Association of the NPC with age, gender and the use of corrective glasses or contact lenses was evaluated by the Student *t* or Mann-Whitney test for parametric and nonparametric data, respectively. Results with a *p* -value ≤ 0.001 were considered statistically significant.

To determine the diagnostic accuracy and optimal cut-off point for the NPC, receiver operating characteristic (ROC) curves were created. This allowed the area under the curve (AUC) to be calculated, along with sensitivity, specificity, and 95% confidence intervals (CIs) associated with these values.

## Results

A total of 50 patients with VM and 50 patients without VM (control group) were included in the study, with a median age of 38 (25–69) years. Table 1 shows the demographic characteristics and NPC of the patients.

**Table 1:** Demographic characteristics and NPC of patients with and without vestibular migraine

Variable	VM group (n = 50)	Control group (n = 50)	*p* -value*
Age (years), mean ± SD	47.00 ± 15.40	38.82 ± 12.85	0.005
Female sex, n (%)	43 (86%)	18 (36%)	–
NPC (cm), mean ± SD	18.50 ± 5.88	8.06 ± 1.46	< 0.001
Test performed with corrective lenses, n (%)	32 (64%)	17 (34%)	< 0.001

**Abbreviations:** NPC, near point of convergence; SD, standard deviation; VM, vestibular migraine. **Note:** *Mann-Whitney test.

Patients' mean age was 38.82, with a standard deviation (SD) of: ± 12.85 years in the control group, and 47.00 ± 15.40 years in the VM group. There was significant difference in age between the groups ( *p* = 0.005).

In the control group, 18 patients were women (36%). The NPC ranged from 5 to 10 cm, with a mean of 8.06 ± 1.46 cm. During the test, 17 (34%) wore corrective glasses or contact lenses.

In the VM group, 43 patients were women (86%). The NPC ranged from 8 to 39 cm, with a mean of 18.50 ± 5.88 cm. During the test, 32 (64%) wore corrective glasses or contact lenses.

**Fig. 1:**
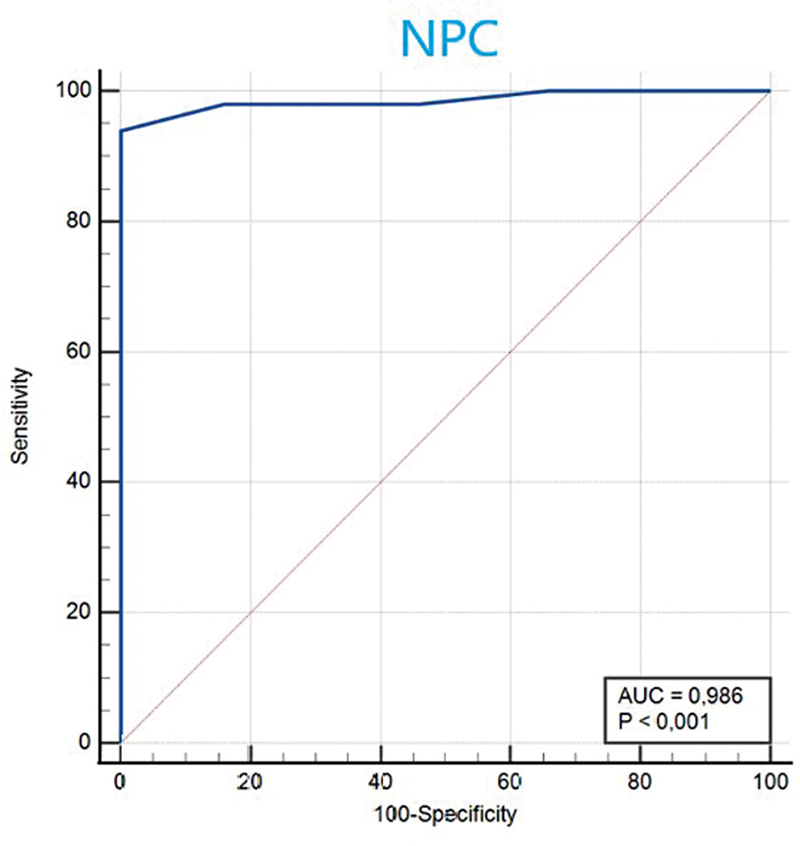
Receiver operating characteristic (ROC) curve for the near point of convergence (NPC) test. **Abbreviation:** AUC, area under the curve.

Therefore, the NPC was significantly higher in patients with VM than in the control group ( *p* < 0.001). The AUC for the NPC test was 0.986 (95% CI: 0.938–0.999; *p* < 0.0001), suggesting that NPC was able to accurately discriminate between patients with and without VM with a sensitivity of 94% and specificity of 100% ( Fig. 1 ). The optimal cut-off point for the NPC was > 10.

The comparison stratified by age (< and ≥ 38 years) and gender showed that the mean NPC was significantly higher in patients with VM compared to the control group ( *p* < 0.001). Likewise, the mean NPC was significantly higher in patients with VM compared to the control group when stratified by use of corrective glasses or contact lenses during the NPC test ( *p* < 0.001), as shown in Table 2 .

**Table 2:** Comparison of the NPC between patients with vestibular migraine and controls stratified by age, use of corrective lenses during the test, and sex

Variable	Group	NPC (cm), mean ± SD	n	*p* -value*
Age < 38 years	Control	8.15 ± 1.37	33	< 0.001
	VM	17.21 ± 6.82	19	
Age ≥ 38 years	Control	7.88 ± 1.65	17	< 0.001
	VM	19.29 ± 5.19	31	
With corrective lenses	Control	8.00 ± 1.41	17	< 0.001
	VM	17.88 ± 5.61	32	
Without corrective lenses	Control	8.09 ± 1.51	33	< 0.001
	VM	19.61 ± 6.35	18	
Female	Control	8.11 ± 1.323	18	< 0.001
	VM	18.30 ± 6.104	43	
Male	Control	8.03 ± 1.555	32	−
	VM	19.71 ± 4.461	7	

**Abbreviations:** NPC, near point of convergence; SD, standard deviation; VM, vestibular migraine. **Note:** *Mann-Whitney test.

## Discussion

Convergence insufficiency is well known as a cause of asthenopia (eye strain) that presents as exophoria, reduced NPC, and/or reduced convergence amplitudes. 6 There are four cases of typical convergence insufficiency associated only with the postmigraine period documented in a case series. 4 Our study showed that all patients of the control group had a normal NPC (< 10 cm) and almost all patients with VM had convergence insufficiency (NPC > 10 cm), except for one patient who had an NPC of 8 cm.

Although there were significant differences in age and gender between groups, when patients were stratified by these characteristics, the NPC was also significantly higher in those of the VM group compared to the control one.

A disrupted balance between the sympathetic and parasympathetic systems can be the pathophysiological mechanism underlying migraine disease. 7 Altered pupillary function has been reported in patients with migraine both during attacks and in the intercritical phase. 8 A recent study described the presence of “pupillary nystagmus,” 9 characterized by cycles of dilation and narrowing of the pupil under constant lighting conditions. This condition indicates a visual dependence typically associated with visually induced vertigo, which is one of the symptoms defined by the classification of vestibular symptoms of the Bárány society, 10 often associated with migraines or VM.

Our theory is that convergence insufficiency in patients with VM may contribute to visually induced vertigo. Only one patient in the VM group had NPC < 10 cm. This finding was expected because distinct mechanisms may be involved in the pathophysiology of migraines/VM. All patients with refractive errors wore corrective lenses or glasses to avoid interference with NPC measurements.

## Conclusion

Our results suggest that convergence insufficiency is a common sign in patients with VM and may be considered a potential clinical biomarker. However, further studies are needed to confirm this hypothesis.
